# Rényi’s Entropy, Statistical Order and van der Waals Gas

**DOI:** 10.3390/e24081067

**Published:** 2022-08-02

**Authors:** Flavia Pennini, Angelo Plastino

**Affiliations:** 1 Departamento de Física, Universidad Católica del Norte, Av. Angamos 0610, Antofagasta 1270709, Chile; fpennini@ucn.cl; 2 Departamento de Física, Facultad de Ingeniería, Universidad Nacional de Mar del Plata (UNMDP), CONICET, Mar del Plata 3350, Argentina; 3 Instituto de Física La Plata—CCT-CONICET, Universidad Nacional de La Plata, C.C. 727, La Plata 1900, Argentina

**Keywords:** Rényi’s entropy, disequilibrium, canonical ensemble, van der Waals

## Abstract

The notion of statistical order derives from the disequilibrium concept introduced by López-Ruiz, Mancini, and Calbet thirty years ago. In this effort, it is shown that the disequilibrium is intimately linked to the celebrated Rényi entropy. One also explores this link in connection with the van der Waals gas description.

## 1. Introduction

In this work, one wishes to link the notion of Rényi’s entropy to that of statistical order. We start then by recalling some essential features of this kind of order.

### 1.1. Statistical Disorder

Statistically, maximum or total disorder is associated with a *uniform probability distribution* (UPD). Thus, the more dissimilar a given probability distribution (PD) is to the UPD, the more statistical order this given PD represents. Recall at this point that the ordinary entropy *S* adequately grasps disorder as well and is maximized by the UPD. Furthermore, recall first of all that the Shannon entropy *S* is a measure of disorder, as extensively discussed in Refs. [1,2]. López-Ruiz, Mancini and Calbet (LMC) [1,2] defined an appropriate tool called disequilibrium *D* to measure statistical order. It is the distance in probability space between an extant PD and the UPD. Furthermore, they baptized the product SD as the statistical complexity *C*

(1) C=SD.

Since one is interested here in the notion of statistical order represented by *D*, from here on, we call this quantity the statistical order index (SOI).

For an N-microstates system, each element of the UPD is equal to 1/N. Thus, if the elements of the extant PD are called $p_{i}$, then *D* is given by [1]

(2) $$D=\sum_{i=1}^{N}\;{\left(p_{i}-\frac{1}{N}\right)}^{2},$$

and the Shannon entropy is

(3) $$S=\sum_{i=1}^{N}\;p_{i}\ln p_{i},$$

where $p_{1},p_{2},\ldots ,p_{N}$ are de corresponding probabilities, with the condition $\sum_{i=1}^{N}\;p_{i}=1$ [1].

In this effort, one will focus attention, within a classical canonical-ensemble environment, on the LMC statistical order index concept and show that it is intimately linked to Rényi’s entropy. One focuses attention upon classical integrable systems and will associate them with an SOI. As a matter of fact, *it will be seen that the SOI (given by D) can entirely replace the partition functions in thermal relationships*.

### 1.2. Rényi’s Entropy

Rényi’s entropy plays a significant role in Information Theory [3]. For example, it generalizes Hartley’s and Shannon–Boltzmann’s entropies and the concept of dimension. It is relevant in ecology and statistics as a diversity index [4,5], as well as a measure of quantum entanglement and much more. It is thus an entropic functional of immense relevance (for more details, see the other articles of this Special Issue).

The Rényi’s entropy of order *q*, with 0≤q<∞ and q≠1, is defined as

(4) $$R_{q}=\frac{1}{1-q}\ln\left(\sum_{i=1}^{N}p_{i}^{q}\right),$$

where $p_{1},p_{2},\ldots ,p_{N}$ are the corresponding set of probabilities [6]. Appealing to the L’Hôpital’s rule, it is possible to demonstrate that, when the limit *q* tends to unity, Rényi’s entropy approaches to Shannon’s entropy *S* (given by Equation (3)), i.e.,

(5) $$S\equiv S_{1}=\lim_{q\to 1}R_{q}.$$

Moreover, the max-entropy or Hartley entropy is the special case of Rényi’s entropy for q=0, i.e.,

(6) $$R_{0}=\lim_{q\to 0}R_{q}=\ln\left(\sum_{i=1}^{N}p_{i}^{0}\right)=\ln N.$$

### 1.3. Review on Properties of the Statistical Order Index

Consider a classical ideal system of identical particles contained in a volume *V* and Hamiltonian $H_{0}\left(r,p\right)$, where (r,p) are the concomitant phase space variables. Furthermore, the system is in equilibrium at temperature *T*. The ensuing distribution becomes [7]

(7) $$\rho_{0}\left(r,p\right)=\frac{e^{-\beta H_{0}\left(r,p\right)}}{Q_{N}^{\left(0\right)}\left(V,T\right)},$$

with $\beta =1/k_{B}T$, and $k_{B}$ the Boltzmann constant.

The canonical partition function is

(8) $$Q_{N}^{\left(0\right)}\left(V,T\right)=\int d\Omega \;e^{-\beta \;H_{0}\left(r,p\right)},$$

with $d\Omega =d^{3N}r\;d^{3N}p/N!h^{3N}$, while Helmholtz’ free energy $A_{0}$ reads [7]

(9) $$A_{0}\left(N,V,T\right)=-k_{B}T\;\ln Q_{N}^{\left(0\right)}\left(V,T\right).$$

López-Ruiz showed in Ref. [8] that the statistical order index $D_{0}\left(N,V,T\right)$ can be cast as

(10) $$D_{0}\left(N,V,T\right)=e^{2\beta \;[A_{0}\left(N,V,T\right)-A_{0}\left(N,V,T/2\right)]},$$

a form valid only for continuous PDs. Now, change *T* to T/2 in Equation (9) and replace the ensuing expression into Equation (10). Thus,

(11) $$D_{0}\left(N,V,T\right)=\frac{Q_{N}^{\left(0\right)}\left(V,T/2\right)}{{\left[Q_{N}^{\left(0\right)}\left(V,T\right)\right]}^{2}}.$$

An alternative D—expression is obtained via definitions (7) and (29), as was shown in Ref. [9], since

(12) $$Q_{N}^{\left(0\right)}\left(V,T/2\right)=\int d\Omega \;e^{-2\beta \;H_{0}\left(r,p\right)}.$$

Therefore, replacing the above expression with Equation (11), one has

(13) $$\begin{array}{ccc} D_{0}\left(N,V,T\right) & = & \int d\Omega \;{\left(\frac{e^{-\beta H_{0}\left(r,p\right)}}{Q_{N}^{\left(0\right)}\left(V,T\right)}\right)}^{2}=\\ & = & \int d\Omega \;(\rho_{0}{\left(r,p)\right)}^{2}, \end{array}$$

an often used expression (see Ref. [10]). Note that one adds the subscript 0 (or superscript (0)) to indicate that the system under consideration is a classical ideal gas. This notation is important to avoid confusion in future sections.

## 2. Statistical Order and Thermodynamic Relations

A reminder that *D* is an expression of statistical order and constitutes an essential concept. In Ref. [9], it has been shown for the classical ideal gas in a canonical ensemble that the basic classical thermodynamic relations (TR) are expressed in statistical order terms via the statistical order index, which we now call $D_{0}\left(N,V,T\right)$. The relevant TR, in addition to Helmholtz’ free energy $A_{0}$, read [7]

(14) $$\begin{array}{ccc} U_{0} & = & -{\left(\frac{\partial \ln Q_{N}^{\left(0\right)}\left(V,T\right)}{\partial \beta }\right)}_{N,V}, \end{array}$$

(15) $$\begin{array}{ccc} \mu_{0} & = & -k_{B}T{\left(\frac{\partial \ln Q_{N}^{\left(0\right)}\left(V,T\right)}{\partial N}\right)}_{V,T}, \end{array}$$

(16) $$\begin{array}{ccc} P_{0} & = & k_{B}T{\left(\frac{\partial \ln Q_{N}^{\left(0\right)}\left(V,T\right)}{\partial V}\right)}_{N,T}, \end{array}$$

(17) $$\begin{array}{ccc} S_{0} & = & k_{B}{\left(\frac{\partial (T\ln Q_{N}^{\left(0\right)}\left(V,T\right))}{\partial T}\right)}_{N,V}. \end{array}$$

One refers above, respectively, to the mean energy $U_{0}$, the chemical potential $\mu_{0}$, pressure $P_{0}$, and entropy $S_{0}$ that in turn depend on the number of particles, volume *V* and temperature *T*. As it was stated in Ref [9], it is possible to recast these TR in terms of the statistical order index represented by $D_{0}$.

Deriving Equation (11) with respect to β, it is possible to find

(18) $$\begin{array}{c} {\left(\frac{\partial \ln D_{0}\left(N,V,T\right)}{\partial \beta }\right)}_{N,V}=\\ ={\left(\frac{\partial \ln Q_{N}^{\left(0\right)}\left(V,T/2\right)}{\partial \beta }\right)}_{N,V}-2{\left(\frac{\partial \ln Q_{N}^{\left(0\right)}\left(V,T\right)}{\partial \beta }\right)}_{N,V}. \end{array}$$

Furthermore, using Equation (14) and placing it into Equation (18), one finds

(19) $${\left(\frac{\partial \ln D_{0}\left(N,V,T\right)}{\partial \beta }\right)}_{N,V}=2\left(U_{0}\left(T\right)-U_{0}\left(T/2\right)\right).$$

The equipartition theorem for the energy states that

(20) $$U_{0}=\frac{f}{2}\;k_{B}T,$$

with *f* the number of degrees of freedom [11,12]. Thus, from (20), one has $U_{0}\left(T/2\right)=U_{0}\left(T\right)/2.$ Inserting Equation (20) into Equation (19), one finds

(21) $${\left(\frac{\partial \ln D_{0}\left(N,V,T\right)}{\partial \beta }\right)}_{N,V}=U_{0}.$$

One emphasizes that (19) is of a more general character than (21) because the latter holds only for systems that satisfy the equipartition of energy. Now, differentiating (11) with respect to $U_{0}$, one sees that

(22) $$\begin{array}{c} {\left(\frac{\partial \ln D_{0}\left(N,V,T\right)}{\partial U_{0}}\right)}_{N,V}=\\ ={\left(\frac{\partial \ln Q_{N}^{\left(0\right)}\left(V,T/2\right)}{\partial U_{0}}\right)}_{N,V}-2{\left(\frac{\partial \ln Q_{N}^{\left(0\right)}\left(V,T\right)}{\partial U_{0}}\right)}_{N,V}. \end{array}$$

Given that *U* is linked to β via Equation (20), one also has $\beta ={\left(\partial \ln Q_{N}^{\left(0\right)}/\partial U_{0}\right)}_{N,V}$.

Then, assuming once more equipartition, one finds

(23) $${\left(\frac{\partial \ln D_{0}\left(N,V,T\right)}{\partial U_{0}}\right)}_{N,V}=-\beta .$$

The pair of relations (21) and (23) constitutes a basic theoretical set and are called reciprocity relations. According to Jaynes, they yield a *complete* description of the thermodynamic properties, here, in statistical order language, for a system described via Gibb’s canonical ensemble [13]. One sees then that Gibb’s canonical ensemble theory can be totally recast in terms of temperature and statistical order—a significant result indeed.

In addition, one comments that Baez pointed out that, while Shannon’s entropy has a strong thermodynamic’ flavor, $R_{q}$ has not quite been completely integrated into the thermal discourse [3]. Here, one wishes to find a natural role for Rényi’s entropy in physics via the notion of statistical order as represented by the statistical order index *D*. This role is related to the free energy, using a parameter (for an arbitrary fixed temperature $T_{0}$) $q=T_{0}/T$ defined as a ratio of temperatures [9]. It was argued in Ref. [3] that, physically, $T_{0}$ is the temperature for which the system automatically has zero free energy.

Therefore, Baez shows in Equation (9) of Ref. [3] that

(24) $$R_{T_{0}/T}\left(T_{0}\right)=-\frac{A_{0}\left(N,V,T\right)-A_{0}\left(N,V,T_{0}\right)}{k_{B}\left(T-T_{0}\right)}.$$

Setting now $T_{0}=T/2$ and employing Equation (10), one finds

(25) $$D\left(N,V,T\right)=e^{-R_{1/2}\left(N,V,T/2\right)},$$

which is the wished-for direct relation between Rényi’s entropy of order 1/2 and the statistical order index *D*. To summarize, it has been connected to Rényi’s entropy with statistical order [9].

Indeed, a possible generalized LMC-like complexity family that involves Rényi’s entropy instead of Shannon’s is given by this expression

(26) $$C_{q}\left(N,V,T\right)=e^{R_{q}\left(N,V,T\right)-R_{1/2}\left(N,V,T/2\right)},$$

as it was pointed out in Ref. [9]. We see that one can also express $C_{q}$ solely in Rényi’s terms. To sum up: statistical order is represented by $R_{1/2}\left(N,V,T/2\right)$.

## 3. Real Gases Application

### 3.1. Notions about Real Gases

Consider now intermolecular interactions in a classical gas of identical particles, confined into a space of volume *V* and in equilibrium at the temperature *T*. The pertinent Hamiltonian reads as a sum over the number of particles *N* [7]. Considering particles *i* and *j*, we have

(27) $$H\left(r,p\right)=H_{0}\left(r,p\right)+\sum_{i<j}\;u_{ij},$$

where $H_{0}\left(r,p\right)=\sum_{i=1}^{N}\left(p_{i}^{2}/2m\right)$ is the free Hamiltonian, and *m* is the mass of the system. Moreover, $u_{ij}=u(|r_{i}-r_{j}\left|\right)$ is the energy of interaction between the particles *i*th and *j*th, a function of the inter-particle distance only. The second sum in the right-hand term of the Hamiltonian runs over the N(N−1)/2 extant pairs of particles [7]. For such a system, the canonical partition function is the product of the ideal gas partition function $Q_{N}^{\left(0\right)}$ and a configuration integral $Z_{N}$ [7]

(28) $$Q_{N}\left(V,T\right)=Q_{N}^{\left(0\right)}\left(V,T\right)\;Z_{N}\left(V,T\right),$$

where, as just stated, the canonical partition function for the ideal gas is

(29) $$Q_{N}^{\left(0\right)}\left(V,T\right)=\int d\Omega \;\exp\left(-\beta \;H_{0}\left(r,p\right)\right)=\frac{1}{N!}{\left(\frac{V}{\lambda^{3}}\right)}^{N},$$

with λ as the particles’ mean thermal wavelength $\lambda =h/\sqrt{2\pi mk_{B}T}$ and *h* the Planck’s constant [7]. The quantity $Z_{N}\left(V,T\right)$ is the well-known configuration integral given by [7,14]

(30) $$Z_{N}\left(V,T\right)=\frac{1}{V^{N}}\;\int d^{3N}r\;\exp\left(-\beta \sum_{i<j}\;u_{ij}\right).$$

### 3.2. Rényi Entropy for a Real Gas

In this article, one focuses attention on the relation of Rényi’s entropy to statistical order via its connection with the free energy, as explained by Baez in Ref. [3]. For such purpose, one first defines Rényi’s entropy of order *q* for a classical system in the following manner

(31) $$R_{q}=\frac{k_{B}}{1-q}\ln\int d\;\Omega \;{\left[\rho \left(r,p\right)\right]}^{q},$$

where dΩ is the differential of the phase space and ρ(r,p) the probability distribution of the system. With the motif of connecting with thermodynamics, one adds the Boltzmann constant to the definition (31). Second, one considers $\rho \left(r,p\right)=\exp\left(-\beta H\left(r,p\right)\right)/Q_{N}\left(V,T\right)$, which is associated with the Hamiltonian (27) of the vdW gas. Therefore, setting

(32) $$X=\frac{1}{h^{3N}N!{\left[Q_{N}\left(V,T\right)\right]}^{q}},$$

one obtains,

(33) $$R_{q}=\frac{k_{B}}{1-q}\ln\left[X\;\int d^{3N}p\;\exp\left(-q\beta H_{0}\left(r,p\right)\right)\;\int d^{3N}r\;\exp\left(-q\beta \sum_{i<j}\;u_{ij}\right)\right].$$

Let us single out the following two terms

(34) $$\int d^{3N}p\;\exp\left(-q\beta H_{0}\left(r,p\right)\right)=\frac{h^{3N}N!}{V^{N}}\;Q_{N}^{\left(0\right)}\left(V,T/q\right),$$

and

(35) $$\int d^{3N}r\;\exp\left(-q\beta \sum_{i<j}\;u_{ij}\right)=V^{N}\;Z_{N}\left(V,T/q\right).$$

Then, replacing Equations (34) and (35) in Equation (33), one is immediately led to the Ré’s entropy given by

(36) $$R_{q}=\frac{k_{B}}{\left(1-q\right)}\ln\left\{\frac{Q_{N}\left(V,T/q\right)}{{\left[Q_{N}\left(V,T\right)\right]}^{q}}\right\}.$$

Via Equation (28), one then rewrites Equation (36) as

(37) $$R_{q}=k_{B}\ln\left\{\frac{1}{N!}{\left(\frac{V}{\lambda^{3}}\right)}^{N}\right\}+\frac{k_{B}}{1-q}\ln\left\{q^{-3N/2}\frac{Z_{N}\left(V,T/q\right)}{{\left[Z_{N}\left(V,T\right)\right]}^{q}}\right\},$$

where one has considered the canonical partition function of the ideal gas given by Equation (29). Note that we have changed *T* by T/q. In addition, one has taken into account the transformation $Q_{N}\left(V,T/q\right)=Q_{N}^{\left(0\right)}\left(V,T/q\right)Z_{N}\left(V,T/q\right)$, with $Q_{N}^{\left(0\right)}\left(V,T/q\right)=q^{3N/2}\;Q_{N}^{\left(0\right)}\left(V,T\right)$.

Using Stirling’s approximation (lnN!≈NlnN−N) and conveniently rearranging terms in Equation (37), one arrives at

(38) $$R_{q}=k_{B}\ln{\left(\frac{V}{N\lambda^{3}}\right)}^{N}+Nk_{B}\left(1-\frac{3}{2}\;\frac{\ln q}{1-q}\right)+\frac{k_{B}}{1-q}\ln\left\{\frac{Z_{N}\left(V,T/q\right)}{{\left[Z_{N}\left(V,T\right)\right]}^{q}}\right\}.$$

A reminder now that the classical entropy of the ideal gas is called the Sackur–Tetrode [14]

(39) $$S_{0}=k_{B}\ln{\left(\frac{V}{N\lambda^{3}}\right)}^{N}+\frac{5Nk_{B}}{2}.$$

Finally, considering Equation (39), one sees that Equation (38) can be also written as

(40) $$R_{q}=S_{0}-\frac{3Nk_{B}}{2}\left(1+\frac{\ln q}{q-1}\right)+\frac{k_{B}}{1-q}\ln\left\{\frac{Z_{N}\left(V,T/q\right)}{{\left[Z_{N}\left(V,T\right)\right]}^{q}}\right\}.$$

### 3.3. Dilute Gas

If the gas density is low enough (n=N/V=1/v≪1), the configuration integral is suitably approximated, as it was shown in Ref. [14]. One usually approximates the interaction $u_{ij}$ above in the form of a suitable function u(r) [14].

(41) $$Z_{N}\left(V,T\right)\approx \exp\left(-n\;N\;B_{2}\left(T\right)\right),$$

where $B_{2}\left(T\right)$ is the second Virial coefficient, given by [14]

(42) $$B_{2}\left(T\right)=-\frac{1}{2}\;\int d^{3}r\;\left[\exp(-\beta u(r))-1\right].$$

In this case, Equation (40) becomes

(43) $$R_{q}=S_{0}-\frac{3Nk_{B}}{2}\left(1+\frac{\ln q}{q-1}\right)+\frac{nNk_{B}}{q-1}\left[B_{2}\left(T/q\right)-qB_{2}\left(T\right)\right].$$

### 3.4. The Van Der Waals Instance

One appeals now to the van der Waals assumptions [9]: u(r)=∞ for $r<r_{o}$, and exp(−βu(r))≈1−βu(r) for $r>r_{o}$, where $r_{o}$ denotes here the minimum possible separation between molecules [14]. It is easy then to compute the second virial coefficient by recourse to Equation (42), which, in this case, the virial coefficient $B_{2}$ becomes

(44) $$B_{2}\left(T\right)=b-\beta a,$$

where $b=2\pi r_{0}^{3}/3$ is related to the volume of a hard-sphere molecular gas, and

(45) $$a=2\pi \int_{r_{0}}^{\infty }\;dr\;r^{2}u\left(r\right),$$

is the mean potential energy, which measures the intermolecular interaction strength. One also sees that

(46) $$B_{2}\left(T/q\right)=b-q\beta a.$$

Therefore, the van der Waals Rényi’s entropy given by Equation (43) becomes

(47) $$R_{q}=S_{vdW}-\frac{3Nk_{B}}{2}\left(1+\frac{\ln q}{1-q}\right),$$

where

(48) $$S_{vdW}=S_{0}-Nk_{B}\left(\frac{b}{v}\right),$$

is the Shannon’s entropy for the van der Waals gas [15]. Notice that, in the limit that *q* tends to unity, the term lnq/(1−q)→−1, then $R_{1}=\lim_{q\to 1}S_{q}=S_{vdW}$.

In addition, one calculates the statistical complexity according to definition (26) and using Equation (48). Thus, one has

(49) $$C_{q}=\exp\left(-\frac{3Nk_{B}}{2}\left(\frac{\ln q}{1-q}+2\ln2\right)\right),$$

which is independent of the temperature *T*. Note that as *N* grows, the system becomes less complex.

**Figure 1:**
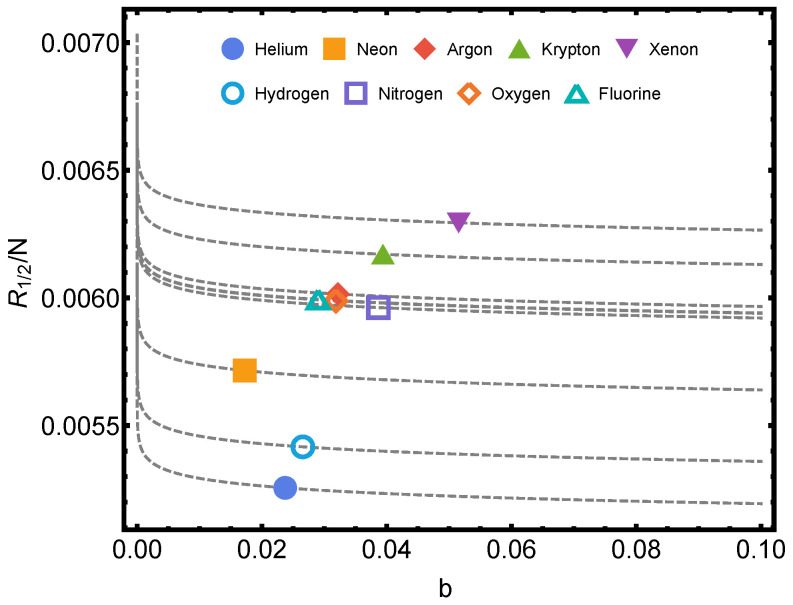
Rényi’s entropy per particle being q=1/2 for the typical van der Waals values $T=T_{c}=8a/\left(27b\right)$ and $v=v_{c}=3b$ versus *b*. One considers the noble gases helium, neon, argon, krypton, and xenon, and also, hydrogen, nitrogen, oxygen, and fluorine. The numerical values of *a* and *b* are given in the table of Ref. [16]. The lines are visual aids representing virtual trajectories as *b* varies.

**Figure 2:**
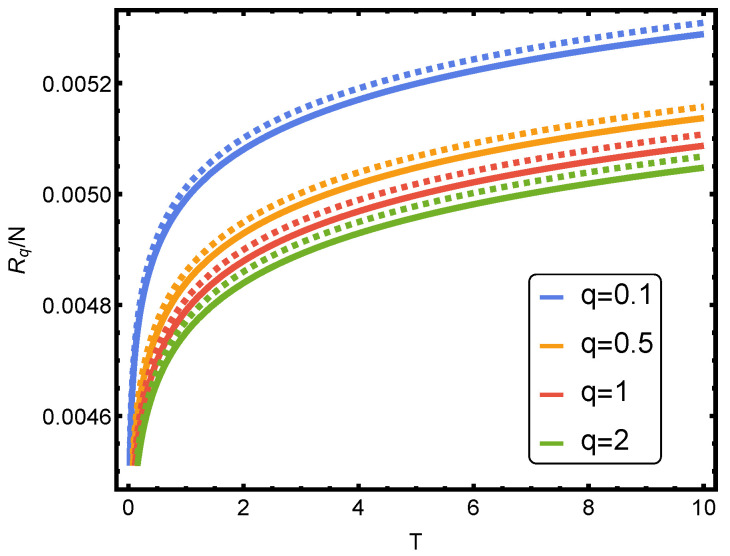
Rényi’s entropy per particle versus temperature *T* for several values of *q*. One takes v=0.0001. The solid curve corresponds to b≠0 (vdW case) and the dashed curve to b=0 (ideal case). The numerical value of *b* is of the helium, obtained in the table of Ref. [16].

In Figure 1, Rényi’s entropy is plotted for q=1/2, corresponding to some noble gases versus the parameter *b* of Equation (44). Here, one evaluates things at the van der Waals critical (1) temperature $T_{c}$ and (2) volume per particle $v_{c}$ [12,15]. Also, we plot the Rényi’s entropy against temperature *T* for several values of parameter *q* in Figure 2.

## 4. Some Relations for the Statistical Order in the vdW Approximation

In this section, one will apply some ideas developed in Section 2. Therefore, one needs to consider the statistical order index *D* in the vdW instance.

In order to repeat the procedure developed in Section 2, one uses the general definition of *D*, which in this case is

(50) $$D\left(N,V,T\right)=\frac{Q_{N}\left(V,T/2\right)}{{\left[Q_{N}\left(V,T\right)\right]}^{2}}=D_{0}\left(N,V,T\right)\;\frac{Z_{N}\left(V,T/2\right)}{{\left[Z_{N}\left(V,T\right)\right]}^{2}}.$$

Taking first the logarithm of *D* and then differentiating with respect to β, one obtains

(51) $${\left(\frac{\partial \ln D}{\partial \beta }\right)}_{N,V}={\left(\frac{\partial \ln D_{0}}{\partial \beta }\right)}_{N,V}+\frac{\partial }{\partial \beta }{\left(\frac{Z_{N}\left(V,T/2\right)}{{\left[Z_{N}\left(V,T\right)\right]}^{2}}\right)}_{N,V}.$$

Considering Equation (41), one arrives at

(52) $${\left(\frac{\partial \ln D}{\partial \beta }\right)}_{N,V}={\left(\frac{\partial \ln D_{0}}{\partial \beta }\right)}_{N,V}+\frac{N}{v}\frac{\partial }{\partial \beta }\left[2B_{2}\left(T\right)-B_{2}\left(T/2\right)\right].$$

Now, taking into account the virial coefficient $B_{2}$ given by Equation (46), one finally obtains

(53) $${\left(\frac{\partial \ln D}{\partial \beta }\right)}_{N,V}={\left(\frac{\partial \ln D_{0}}{\partial \beta }\right)}_{N,V}=U_{0},$$

where the last equality is due to Equation (21).

The result (53) can also be achieved via the statistical order obtained in Ref. [15], whose appearance is

(54) $$D\left(N,V,T\right)=D_{0}\left(N,V,T\right)\;\exp\left(Nb/v\right).$$

One must emphasize that the derivative of the vdW lnD with respect to β is not equal to the energy of the ideal gas. This is because the term $2B_{2}\left(T\right)-B_{2}\left(T/2\right)=b$ does not depend on *T* and then ∂b/∂β=0. Note also the vdW Hamiltonian is not quadratic, unlike that for the ideal gas.

## 5. *R_q_*—Related Statistical Complexity via Fisher’S Information Measure

The statistical complexity can be thought of as the product of a measure of order times a measure of disorder (the entropy). Accordingly, one considers now, for thee sake of completeness, an alternative, Rényi related, definition of the statistical complexity (26) for the case b=0 (ideal gas) as follows

(55) $$C_{q}=I_{p}\;R_{q},$$

where $I_{p}$ is the Fisher information measure [17]. This quantity is a measure of order [17], as *D*, so that it can legitimately replace *D* in the $C_{q}$ definition. $I_{p}$ in the phase-space appearance appears as

(56) $$I_{p}=\int d\Omega \;\rho_{0}\left(r,p\right)\;\sum_{i=1}^{3N}{\left(\frac{\partial \ln\rho_{0}\left(r,p\right)}{\partial p_{i}}\right)}^{2},$$

where the probability distribution $\rho_{0}\left(r,p\right)=\exp\left(-\beta H_{0}\left(r,p\right)\right)/Q_{N}^{\left(0\right)}$, and $R_{q}$ is Rényi’s entropy obtained in Equation (47). Performing the above integral, one can analytically obtain (this is the great advantage of this $C_{q}$)

(57) $$I_{p}=\frac{3N}{mk_{B}T}.$$

**Figure 3:**
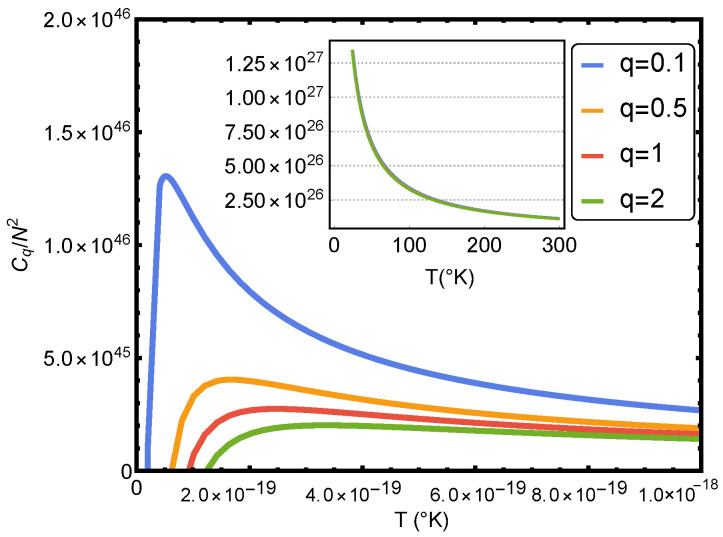
Statistical complexity $C_{q}/N^{2}$ versus the temperature *T* for several values of *q*. One considers v=2 and b=0 (ideal case).

One appreciates the shape of $C_{q}$ in Figure 3. The complexity $C_{q}$ reaches its maximum value near T=0 and approaches zero as *T* approaches infinity, as one sees in the figure. Of course, the complexity diminishes as *T* augments and grows when the particle number increases (see Figure 3). The complexity grows as *q* decreases. Its maximum corresponds to temperatures for which the classical treatment is no longer valid. This last statement is carefully investigated in Ref. [15].

## 6. Conclusions

In this paper, one has connected Rényi’s entropy (RE) with the statistical order–disorder disjunction. Disorder is represented by several types of entropies, Rényi’s being one of them. Order is represented by Fisher’s information measure and also by high values of different types of distances in probability space from incumbent probability distribution to the uniform one (PU). The Euclidean distance is called the disequilibrium *D*. The Kullback–Leibler divergences involving the PU are another possibility.

Equation (25) directly links *D* with the q=1/2 Rényi’s entropy, which gives this special entropy a privileged role in the order–disorder disjunction. This equation straightforwardly states that the degree of order/disorder (O/D) links, at the same time, RE to *D*. This jointly expresses the same O/D situation. One has chosen de van der Waals gas to illustrate some O/D instances in an actual system: several gases.

Accordingly, one has shown in this review note that, in a classical phase space context, with continuous probability distributions, the LMC notion of statistical order index *D* has a suitable role in statistical thermodynamics by virtue of Equation (25), plus a host of other thermal relations that have been discussed above.

Indeed, for the classical ideal gas, in the new thermal relations, the partition function $Q_{N}$ does not need to appear at all, so that one may be tempted to suggest that it has been replaced by the statistical order index. All important thermodynamic relations can indeed be expressed in terms of *D*. One might argue that the logarithm of *D* exhibits properties analogous to those of Massieu potentials. Finally, *D* itself can be simply expressed in terms of a particular Rényi’s entropy. This is not exactly so for the vdW gas because its Hamiltonian is not quadratic.

Interesting future work and open problems would be to apply the exposed ideas considering other information quantifiers, for example, (1) Tsallis entropy, (2) gas equations different from the van der Waals one, or (3) alternative statistical quantifiers. 

## Data Availability

The data presented in this study are available in the article.
